# Genetic architecture and candidate gene identification for grain size in bread wheat by GWAS

**DOI:** 10.3389/fpls.2022.1072904

**Published:** 2022-11-30

**Authors:** Haitao Yu, Yongchao Hao, Mengyao Li, Luhao Dong, Naixiu Che, Lijie Wang, Shun Song, Yanan Liu, Lingrang Kong, Shubing Shi

**Affiliations:** ^1^ College of Agriculture, Xinjiang Agricultural University, Urumqi, Xinjiang, China; ^2^ Wheat Research Institute, Weifang Academy of Agricultural Sciences, Weifang, Shandong, China; ^3^ State Key Laboratory of Crop Biology, Shandong Key Laboratory of Crop Biology, College of Agronomy, Shandong Agricultural University, Taian, China

**Keywords:** wheat, grain size, mapping, GWAS, yield

## Abstract

Grain size is a key trait associated with bread wheat yield. It is also the most frequently selected trait during domestication. After the phenotypic characterization of 768 bread wheat accessions in three plots for at least two years, the present study shows that the improved variety showed significantly higher grain size but lower grain protein content than the landrace. Using 55K SNP assay genotyping and large-scale phenotyping population and GWAS data, we identified 5, 6, 6, and 6 QTLs associated with grain length, grain weight, grain area, and thousand grain weight, respectively. Seven of the 23 QTLs showed common association within different locations or years. Most significantly, the key locus associated with grain length, *qGL-2D*, showed the highest association after years of multi-plot testing. Haplotype and evolution analysis indicated that the superior allele of *qGL-2D* was mainly hidden in the improved variety rather than in landrace, which may contribute to the significant difference in grain length. A comprehensive analysis of transcriptome and homolog showed that *TraesCS2D02G414800* could be the most likely candidate gene for *qGL-2D*. Overall, this study presents several reliable grain size QTLs and candidate gene for grain length associated with bread wheat yield.

## Introduction

Bread wheat is one of the major crops, accounting for nearly 20% of calories in our diet (http://faostat.fao.org). Improvement of yield remains a challenge under heavy population pressure and projected global change (Ray et al., 2013). Grain size is a major determinant of grain weight, besides the number of panicles per plant and the number of grains per panicle (Fan et al., 2006). In wheat breeding, grain size is usually evaluated by grain weight, which is positively correlated with grain length, grain width and grain thickness (Evans, 1972; Fan et al., 2006). Thus, it is vital to identify and introduce favorable genes or alleles controlling grain traits to improve the grain yield in bread wheat breeding.

Using linkage mapping, hundreds of grain size quantitative trait loci (QTLs) have been identified in the past few years (Zhang et al., 2018; Mora-Ramirez et al., 2021; Guo et al., 2022). Recently, multiple signals associated with grain size were detected in different populations *via* genome-wide association study (GWAS) (Breseghello and Sorrells, 2006a; Breseghello and Sorrells, 2006b; Pang et al., 2020). These QTLs are distributed on all the 21 chromosomes of bread wheat. However, the real genes underlying these QTLs have yet to be identified due to the complexity of parental mapping, QTL effect, QTL × genotype and QTL × QTL interactions. Using homology cloning, several orthologous genes associated with grain traits have been isolated and characterized in bread wheat. For instance, TaGW2 and TaGS5 were isolated in wheat based on *OsGW2* and *OsGS5* orthologs in rice (Wang et al., 2016; Zhai et al., 2018). *TaGW2* is involved in regulation of grain weight and grain number in bread wheat (Zhai et al., 2018). *TaGS5* is associated with thousand grain weight (Wang et al., 2016), *TaGW8* is related to grain size in bread wheat (Yan et al., 2019). It is still hard to determine the variation in natural elite alleles of these known genes that can be used in marker assisted selection (MAS) of bread wheat. Therefore, it is still very important to explore and identify new QTLs and their natural allelic variation in wheat breeding.

In this study, we constructed a GWAS panel with 768 bread wheat accessions. After phenotypic evaluation in multiple plots for several years, we performed GWAS to the identify grain size of QTLs. A total of 23 grain size QTLs were identified. For a major grain length QTL *qGL-2D*, we investigated the signatures of natural variation *via* comprehensive analysis of haplotype and evolutionary features. Finally, one candidate gene associated with *qGL-2D* was identified. The results suggest that grain size QTLs and grain length candidate genes as well as information may facilitate MAS of these loci/genes in breeding high-yield wheat in the future.

## Materials and methods

### Materials

A total of 768 bread wheat accessions were used to identify QTLs of grain size, including 683 Chinese resources (560 improved varieties and 123 landraces) and 85 introduced accessions. Field experiments were performed at three locations, i: the Shandong Agricultural University Agronomy Experimental Station in Tai’an from 2016 to 2019, ii: Weifang Academy of Agricultural Sciences in Weifang in 2019, and iii: Jining Academy of Agricultural Sciences in Jining in 2019. Each accession was planted in five-row plots with 5 cm distance between plants and 25 cm distance between rows. The interval between adjacent plots was 50 cm. At the mature stage, we harvest 10 spikes without any mechanical damage, disease or insect infestation. After threshing, we measured thousand grain weight (TGW), grain length (GL), grain width (GW), grain area (GA), grain perimeter (GP), grain roundness (GR), grain diameter (GD), length-to-width ratio (LWR), grain protein content (GPC) and grain starch content (GSC) for each accession using a Crop Grain Appearance Quality Scanning Machine (SC-E, Wanshen Technology Company, Hangzhou, China).

### Genotyping

Genomic DNA was extracted from the seedling leaves of all 768 wheat accessions, followed by further genotyping *via* an Illumina 55K assay. Finally, a total of 47,743 of 53,063 SNPs were identified in the wheat panel. We estimated the whole-genome distribution and minor allele frequency (MAF) of these SNPs using an in-house Python script. Additionally, we performed quality control of SNPs to exclude those with high missing rate (> 50%) and low MAF (< 5%) for further analysis.

### Population structure

We first extracted 45,298 SNPs with miss rate ≤ 0.5 and MAF ≥ 0.05 from 53,063 SNPs using an in-house Python script. Using PLINK (window size 50, step size 50, *r* ^2^ ≥ 0.3), a total of 4,360 independent SNPs were further screened out based on *r* ^2^ of LD ≤ 0.3 (Purcell et al., 2007). The software STRUCTURE was used to calculate varying levels of K (K = 1-20) (Pritchard et al., 2000). We also performed principal component analysis (PCA) and kinship analysis using these independent SNPs and GAPIT software (Lipka et al., 2012; Tang et al., 2016). The phylogenetic analysis of *qGL-2D* was performed by generating a neighbor-joining tree using Mega 7 (Kumar et al., 2016).

### Association mapping

Only 45,298 un-imputed SNPs with miss rate ≤ 0.5 and MAF ≥ 0.05 were used to conduct GWAS for GL, GW, GA and TGW, respectively. The first three PCs were used to construct the PC matrix. We performed GWAS with a Compressed Mixed Linear Model (CMLM) *via* PCA and kinship analysis using default settings of GAPIT (Lipka et al., 2012; Tang et al., 2016). Additionally, the threshold to determine significant association was set at 1.0 × 10^-5^ after Bonferroni-adjusted correction (Pang et al., 2020).

### Expression analysis and epidermal cell observation

Gene expression data from different wheat cultivars were used to analyze the gene expression profiles of the candidate region. Expression data were download from wheat-URGI website (https://wheat-urgi.versailles.inra.fr/Seq-Repository/Expression). Then the transcriptomic information of candidate genes were exacted by a custom python script. Epidermal tissues were peeled off using tweezers under a stereomicroscope. Then, the cell layers were stained with safranin and mounted on glass slides (Matsunami Glass Ind., Japan). The tissue specimens were subjected to observation with a light microscope (BX50F Olympus Optical Co., Ltd, Japan).

### Screening of candidate genes for *qGL-2D*

In order to identify candidate genes for *qGL-2D*, LD heatmaps surrounding peaks were constructed using the R package “LD heatmap” (Shin et al., 2006). Using pairwise LD correlation (*r* ^2^ > 0.6), we mined the candidate regions of *qGL-2D* (Yano et al., 2016). We further investigated the expression of these candidate genes in bread wheat grain using typical materials belonging to different haplotypes.

## Results

### Population structure and grain characterization of 768 bread wheat accessions

**Figure 1:**
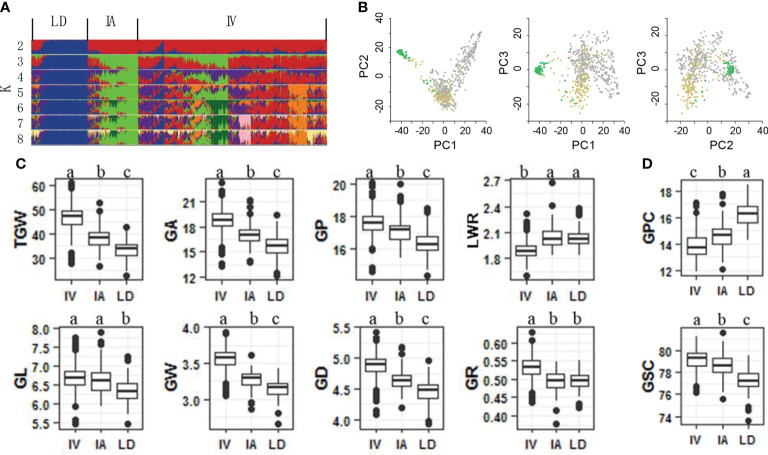
Genetic architecture and characteristic of grain size and grain quality of 768 bread wheat accessions. **(A)** Genetic structure of the panel analyzed using the program STRUCTURE. Landrace (LD), improved variety (IV) and introduced variety (IA) groups appeared at K = 3. **(B)** Principle components analysis reveals that the first 3 principle components explain 17.09%, 6.15% and 3.38% of the genetic variance within the panel. Comparison of grain size traits **(C)** and grain quality traits **(D)** among LD, IA, IV. Different letters above the boxes indicate significant differents (*p* < 0.05) when analyzed by Duncan’s test.

To identify genetic loci associated with grain weight, a panel of 768 bread wheat accessions were constructed, including 560 improved varieties, 123 landraces and 85 introduced accessions. Using a 55K SNP assay, we obtained 47,743 SNPs of the panel. Subsets of these data were further filtered and used in additional analyses (**Figure S1**). A reasonable assessment of population structure facilitates the identification of real marker-trait associations (Crowell et al., 2016; Juliana et al., 2019). Therefore, we calculated varying levels of K means using un-imputed SNPs and STRUCTURE software (Golbeck, 1987). Landrace, improved and introduced varieties appeared clearly at K = 3 (**Figure 1A**). Further PCA indicated that top three PCs accounted for 17.09%, 6.15% and 3.38% of genetic variation within the bread wheat panel (**Figure 1B**). The results suggested obvious genetic differentiation between landrace and improved varieties of bread wheat.

**Figure 2:**
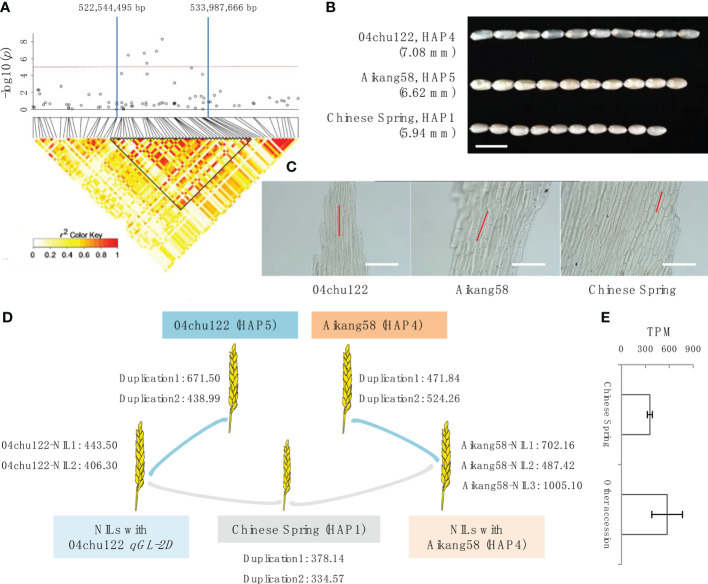
Identification and haplotype analysis of grain length QTL *qGL-2D*. Quantile-quantile (Q-Q) plot **(A)** and manhattan plot **(B)** using 7 groups of GL data of multi-year and multi-plots. *qGL-2D* is an association signal detected in all tests. **(C)** *qGL-2D* haplotype analysis and comparisons of grain length (GL) among four *qGL-2D* haplotypes. **(D)** Comparison of grain area (GA), grain perimeter (GP), grain width (GW), length-width ratio (LWR), grain diameter (GD), grain roundness (GR), hundred grain weight (HGW) and grain protein content (GPC) among four *qGL-2D* haplotypes. For better chart presentation, TGW is replaced by HGW. **(E)** Phylogenetic tree of the four *qGL-2D* haplotypes. The number of landrace (LD), improved variety (IV) and introduced variety (IV) are marked for four haplotypes.

A total of 10 traits were identified in three different plots for two years, including eight grain shape components (TGW, GL, GW, GA, GP, GR, GD, and LWR) and two grain quality components (GPC and GSC). All traits showed high heritability from 89.30% (GSC) to 95.27% (TGW) (**Table S1**). After obtaining the best linear unbiased prediction (BLUP) of each accession with respect to each trait across all traits, the coefficient of variation (CV) of all traits ranged from 1.44% GSC to 15.48% TGW (**Table S1**). GPC was proved to be negatively correlated with the eight grain size components, suggesting that larger, heavier and longer bread wheat grains usually had lower GPC (**Figure S2**). During the domestication of landrace to improved variety, bread wheat grains increased in size, weight, and length, but their GPC decreased (**Figures 1C, D**, **2C**).

### Identification of grain shape QTLs by GWAS

Focusing on four key grain shape traits (GL, GW, GA and TGW), GWAS was performed to identify QTLs based on their respective multi-year and multi-location data and BLUP. A total of 23 QTLs were detected on 12 chromosomes, including 5, 6, 6 and 6 QTLs for GL, GW, GA and TGW, respectively (**Table 1** and **Figures S3**, **S4**). Seven of 23 QTLs showed common association within different locations or years, including *qGW-2B*, *qGL-2D*, *qGW-2D.1*, *qTGW-4A*, *qTGW-5A.1*, *qGA-6D*, *qTGW-6D* and *qTGW-7D*. Consistent with the positive correlations between GL, GW, GA and TGW (**Figure S2**), close linkage, and overlapping or one-factor-to-many-effects (pleiotropy) were detected on chromosome 2D (for *qGA-2D* and *qGL-2D*), chromosome 5A (for *qGA-5A*, *qTGW-5A.1* and *qGL-5A.1)*, chromosome 6D (for *qGA-6D* and *qTGW-6D)*, and chromosome 7D (for *qGA-7D*, *qGW-7D* and *qTGW-7D* (**Table 1**).

**Table 1:** QTL identified for grain weight or shape by combined analysis of six environments and BLUP.

Chr.	QTL	Trait	Environments	Peak SNP	Position	-log10(p)	QTL reported
1D	*qGA-1D.1*	GA	18T	AX-109817000	79999712	5.13	–
	*qGA-1D.2*		19T	AX-86164003	95559483	5.79	–
2A	*qGW-2A*	GW	19T	AX-109994744	721725535	5.56	Wang et al. (2012)
			BLUP	AX-109994744	721725535	5.65	
2B	*qGW-2B*		18T	AX-108936154	720581605	5.45	Zanke et al. (2015)
			19T	AX-108936154	720581605	5.53	
			BLUP	AX-108936154	720581605	5.89	
2D	*qGA-2D*	GA	20W	AX-108767381	528101770	5.29	–
			BLUP	AX-108767381	528101770	5.27	–
	*qGL-2D*	GL	17T	AX-110982403	525904353	6.41	–
			18T	AX-108767381	528101770	6.60	–
			19T	AX-108767381	528101770	8.63	–
			20J	AX-108767381	528101770	6.99	–
			20T	AX-108767381	528101770	7.00	–
			20W	AX-108767381	528101770	6.09	–
			BLUP	AX-108767381	528101770	8.31	–
	*qGW-2D.1*	GW	17T	AX-109910122	587284788	6.01	Ramya et al. (2010)
			18T	AX-94632592	593270570	6.13	
			19T	AX-109464110	585470933	5.87	
			20J	AX-109449735	590677250	6.79	
			20T	AX-94632592	593270570	6.70	
			20W	AX-111098468	593217154	5.99	
			BLUP	AX-111098468	593217154	7.06	
	*qGW-2D.2*		18T	AX-111956072	34428803	6.10	Wang et al. (2019a)
3D	*qGW-3D*		20T	AX-111624595	572830156	5.18	Ma et al. (2019)
4A	*qTGW-4A*	TGW	18T	AX-108908317	681180867	5.13	Zanke et al. (2015)
			19T	AX-108908317	681180867	5.22	
			BLUP	AX-108908317	681180867	5.30	
4B	*qGL-4B*	GL	20T	AX-110919438	643312159	5.69	–
5A	*qGA-5A*	GA	18T	AX-111136203	430037627	5.34	Cheng et al. (2017); Wu et al. (2015).
	*qTGW-5A.1*	TGW	19T	AX-110508884	428416559	5.02	
			20W	AX-110508884	428416559	5.15	
	*qGL-5A.1*	GL	18T	AX-111136203	430037627	5.14	
	*qGL-5A.2*		20J	AX-108762108	595372901	5.32	Wang et al. (2019b)
	*qTGW-5A.2*	TGW	20J	AX-109504344	704583912	5.25	Zanke et al. (2015)
5B	*qTGW-5B*		20T	AX-110427093	34285686	5.09	Yang et al. (2020)
5D	*qGL-5D*	GL	20W	AX-110985437	404832095	5.13	–
6D	*qGA-6D*	GA	17T	AX-110007215	93614544	5.12	Lopes et al. (2013), McCartney et al. (2005), Shi et al. (2017)
			18T	AX-110007215	93614544	6.82	
			20J	AX-110007215	93614544	5.60	
			20W	AX-110007215	93614544	5.87	
			BLUP	AX-110007215	93614544	5.79	
	*qTGW-6D*	TGW	17T	AX-110007215	93614544	5.52	
			18T	AX-110007215	93614544	8.46	
			19T	AX-110007215	93614544	6.49	
			20W	AX-110007215	93614544	6.03	
			BLUP	AX-110007215	93614544	6.26	
7D	*qGA-7D*	GA	20T	AX-110826147	65503524	5.60	Liu et al. (2014), Tang et al. (2017)
	*qGW-7D*	GW	20T	AX-110826147	65503524	5.55	
	*qTGW-7D*	TGW	20T	AX-110826147	65503524	5.87	
			18T	AX-111843581	67448018	5.25	

To validate the results of GWAS, we compared the localization of the QTLs identified in this study with previously detected QTLs associated with bi-parental mapping population. Twelve of 23 QTLs in this study were co-localized with previously reported QTLs, including 1, 6, 3 and 6 QTLs for GL, GW, GA, and TGW, respectively (**Table 1**). The *qGA-6D* and *qTGW-6D* were detected most frequently (five times), followed by *qGA-5A*, *qTGW-5A.1*, *qGL-5A.1*, *qGL-5A.2*, *qGA-7D*, *qGW-7D* and *qTGW-7D* (twice), whereas *qGW-2A*, *qGW-2B*, *qGW-2D.1*, *qGW-2D.2*, *qGW-3D*, *qTGW-4A*, *qGL-5A.2*, *qTGW-5A.2* and *qTGW-5B* were detected rarely (once). Additionally, we also identified six new grain size QTLs, including *qGA-1D.1*, *qGA-1D.2*, *qGA-2D*, *qGL-2D* and *qGL-4B*.

### Haplotype analysis of *qGL-2D*

**Figure 3:**
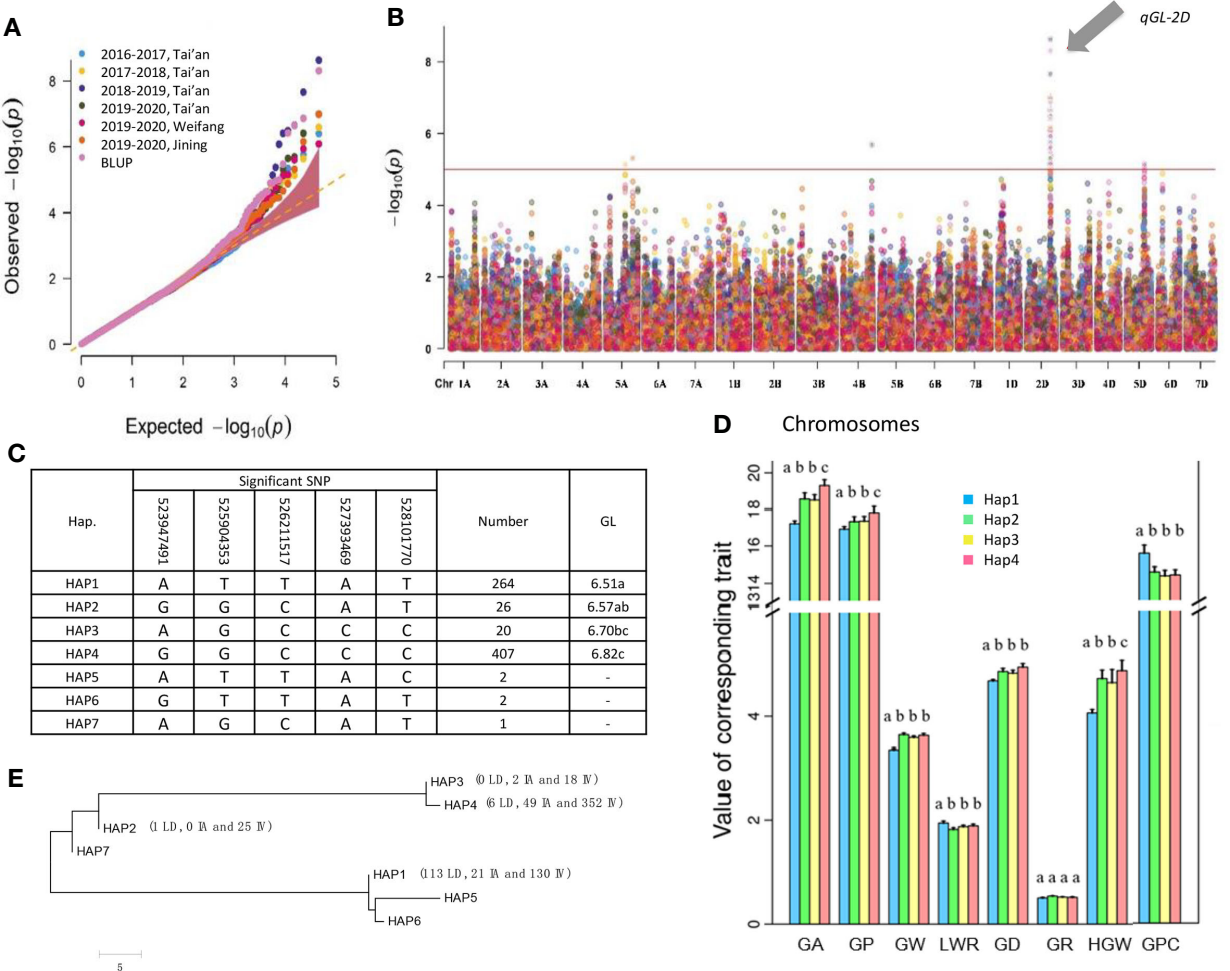
Determination of candidate genes within *qGL-2D*. **(A)** Association signals (top) and LD heatmap (bottom) of *qGL-2D*. Triangular block shows region with strong local LD (*r* ^2^ > 0.6). **(B)** Grain length of 04chu122, Aikang58 and Chinese Spring. Scale bar, 10 mm. **(C)** Epidermal cell length of 04chu122, Aikang58 and Chinese Spring. Scale bars, 200 um. **(D)** Expression level of TraesCS2D02G414800 in 04chu122, Aikang58, Chinese Spring and NILs carried 04chu122 *qGL-2D* or Aikang58 *qGL-2D*. **(E)** Comparison of expression level of Chinese Spring (HAP1), and the other accessions including 04chu122 (HAP5), Aikang58 (HAP4) and NILs carried 04chu122 *qGL-2D* and Aikang58 *qGL-2D*.

The *qGL-2D* was a key locus for GL, as it was detected using the data for each location every year and BLUP (**Figures 3A, B** and **Figure S3**). Using BLUP of GL yielded five significant SNPs (-log(*p*) > 5) representing *qGL-2D*. Thus, the five SNPs were identified *via qGL-2D* haplotype analysis. A total of seven haplotypes were detected, including two high-frequency haplotypes (HAP1 and HAP4, 36.6% and 56.4%), two low-frequency haplotypes (HAP2 and HAP3, 3.6% and 2.8%) and three rare haplotypes (HAP5-7, < 1%) (**Figure 3C**). Among them, GL was the shortest in HAP1 (6.56 mm), followed by HAP2 (6.57 mm) and HAP3 (6.70 mm), whereas HAP4 had the longest GL (**Figure 3C**). For other five traits were related to grain shape (GA, GW, GD and HGW) and grain quality (GPC). The HAP4 exhibited the greatest GA, GW, GD, and HGW, and the lowest GPC (**Figure 3D**). The results suggested that *qGL-2D* was widely involved in grain shape and grain quality.

To determine the evolutionary features of *qGL-2D*, we conducted a phylogenetic analysis of the seven haplotypes. Two major clades were formed (**Figure 3E**). One clade contained a widely divergent group, including HAP4, HAP3, HAP2 and HAP7, the most prevalent haplotypes associated with improved varieties of bread wheat. Another major haplotype in bread wheat landrace, HAP1, was clustered in the other clade (**Figure 3E**). In summary, the *qGL-2D* allele associated with improved varieties of bread wheat showed substantial genetic differences compared with bread wheat landrace, which could be attributed to selective effects on large grain during the process of modern bread wheat improvement.

### Determination of candidate genes within *qGL-2D*

To analyze the candidate gene within *qGL-2D*, we defined the QTL region based on local LD. As indicated in the LD heatmap, an interval from 522,544,495 to 533,987,666 bp on chromosome 2D was an LD block with *r* ^2^ > 0.6 (**Figure 2A**). The *qGL-2D* contains 125 annotated genes. To further reduce the candidate number, we performed transcriptome analysis using one short-grain accession (Chinese Spring (HAP1)), two long-grain bread wheat accessions (Aikang 58 (HAP4), 04chu122 (HAP5)) and 5 BC_2_ near isogenic lines (NILs) carrying *qGL-2D* 04chu122 or aikang58 segment (**Figure 2B**). A total of 29 expressed genes were identified in eight accessions mentioned above (**Table S2**), and only *TraesCS2D02G414800* showed higher expression within two long-grain and eight NILs than in one short-grain accession (**Figures 2D**, **E**). Homology analysis showed that *TraesCS2D02G414800* encodes oleosin, which is involved in seed maturation and germination. Taken together, the results provide possible key candidates for further investigation of the molecular mechanism underlying GL within bread wheat.

## Discussion

Grain size is one of the most frequently selected traits during domestication (Meyer and Purugganan, 2013; Zuo and Li, 2014). Among the many yield-related traits, increased grain size is the main factor associated with increased grain yield at a certain stage of domestication (Zheng et al., 2011). The grains of wild relatives are usually small and round in shape, and domestication has greatly increased the diversity of grain shape and size together with other changes (Fan et al., 2006). Grain size is predominantly determined by genetic factors, whereas grain filling is controlled by both genetic and environmental factors (Sakamoto and Matsuoka, 2008). Our study validated the significant changes in grain size of landrace to improved variety of bread wheat, and also suggested further accumulation of large-size alleles within improved variety rather than landrace. The most significant finding of the present study was the key locus for GL, *qGL-2D*, which showed the highest association after years of multi-plot testing. Haplotype and evolution analysis indicated that the superior allele of *qGL-2D* was mainly hidden in the improved variety rather than in landrace, which may result in significant difference in GL. Identification of the differential expression yielded a single candidate gene of *qGL-2D*. The results provide the opportunity for the delineation of the regulatory mechanism and related processes during grain development.

The coordination of grain size (weight) and grain quality is a major goal in breeding, as the increased grain size often reduces grain quality (Sakamoto and Matsuoka, 2008; Wang et al., 2012). Correlations between traits are a common biological phenomenon, especially those associated with determination of spike, growth duration, yield, and root and shoot (Crowell et al., 2016; Li et al., 2018; Zhao et al., 2019; Zhao et al., 2021). The present study indicated that the grain size increased while the GPC of bread wheat decreased from landrace to improved variety. The long-grain allele of *qGL-2D* showed a lower GPC, while the short-grain allele of *qGL-2D* showed a higher GPC. Pleiotropy and LD in natural population are usually considered as the main factors underlying this phenomenon, which is a major challenge in future breeding programs (Chen and Lübberstedt, 2010; Crowell et al., 2016). The role of two complementary genes associated with grain yield and grain quality requires further analysis (Zuo and Li, 2014).

## Data availability statement

The data presented in the study are deposited in the OMIX repository (https://ngdc.cncb.ac.cn/omix/), accession number OMIX002373.

## Author contributions

S.B.S. and L.R.K. designed and supervised the work; H.T.Y., M.Y.L., L.H.D., N.X.C., L.J.W., S.S. and Y.N.L. performed the research; H.T.Y. and Y.C.H. analyzed the data; H.T.Y. and S.B.S. wrote the paper. All authors read and approved the final manuscript.

## Funding

This work was supported by the Natural Science Foundation of Shandong Province (ZR2020MC096 and ZR2021ZD31).

## Conflict of interest

The authors declare that the research was conducted in the absence of any commercial or financial relationships that could be construed as a potential conflict of interest.

## Publisher’s note

All claims expressed in this article are solely those of the authors and do not necessarily represent those of their affiliated organizations, or those of the publisher, the editors and the reviewers. Any product that may be evaluated in this article, or claim that may be made by its manufacturer, is not guaranteed or endorsed by the publisher.
