# Supplementary material for: Genetic architecture and candidate gene identification for grain size in bread wheat by GWAS
Source: Front Plant Sci. 2022 Nov 30;13:1072904. doi: 10.3389/fpls.2022.1072904 (PMC9748340; doi:10.3389/fpls.2022.1072904)
Supplement: Supplementary file 2 [file DataSheet_2.docx]

**Fig. S1 A summary of SNPs quality of 768 bread wheat accessions. (a)** Number of SNPs with different minor allele frequencies. **(b)** whole-genome distribution of 47,743 SNPs on level of chromosomal window. Window size is 10Mb.

**Fig. S2 Pairwise phenotype correlations.**

**Fig. S3 Quantile-quantile (Q-Q) plot (left) and manhattan plot (right) using 6 groups of grain length data of different years or different plots and its BLUP.**

**
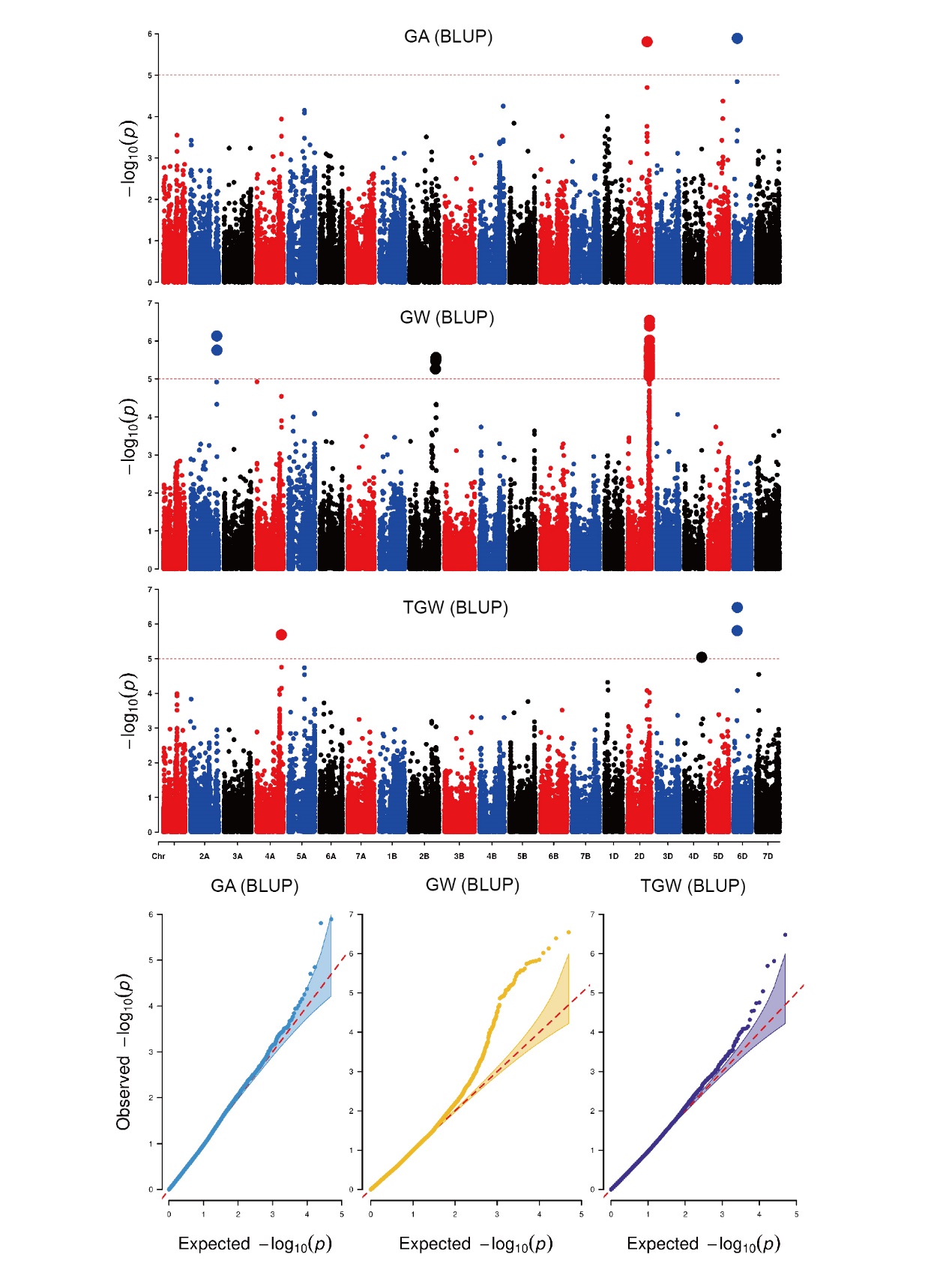
**

**Fig. S4 Quantile-quantile (Q-Q) plots and manhattan plots using BLUPs of grain .**
